# How Competent are Adolescent Bullying Perpetrators and Victims in Mastering Normative Developmental Tasks in Early Adulthood?

**DOI:** 10.1007/s10802-017-0316-3

**Published:** 2017-06-08

**Authors:** Tina Kretschmer, René Veenstra, Susan Branje, Sijmen A. Reijneveld, Wim H. J. Meeus, Maja Deković, Hans M. Koot, Wilma A. M. Vollebergh, Albertine J. Oldehinkel

**Affiliations:** 1 Faculty of Behavioural and Social Sciences, Department of Pedagogy, University of Groningen, Grote Rozenstraat 38, 9712 TJ Groningen, Netherlands; 2 Utrecht University, Utrecht, Netherlands; 3 University Medical Centre Groningen, Groningen, Netherlands; 4 Utrecht University & Tilburg University, Utrecht, Netherlands; 5 Vrije Universiteit Amsterdam, Amsterdam, Netherlands

**Keywords:** Bullying-perpetration, Bullying-victimisation, Developmental tasks, Parental support

## Abstract

A substantive body of literature suggests that those involved in bullying as perpetrators but particularly victims are at greater risk for psychological maladjustment. In comparison, relatively little is known about associations between bullying-victimization and perpetration and mastery of early adult tasks in domains including romantic relationships, education, work, financial competence, and conduct. These links were tested using data from two Dutch cohorts (RADAR-young, *n* = 497, 43% girls; TRAILS, *n* = 2230, 51% girls) who reported on victimization and perpetration at age 11 (TRAILS) and 13 (RADAR-young) and mastery of developmental tasks in early adulthood. Unadjusted regression analyses suggested for both cohorts that perpetrators were less likely to abide the law and more likely to smoke. Victims in TRAILS were less competent in the domains of education, work, and finances, and more likely to smoke in RADAR-young. Adjusting for childhood demographics and child intelligence and including psychopathology in the prediction models substantially reduced the strength of associations between bullying involvement and later outcomes in both cohorts; although association were retained between victimization and welfare dependence and perpetration and crime involvement in TRAILS. Parental support did not buffer associations in either sample and neither were gender differences detected. Overall, findings underline that negative outcomes of bullying are not only a concern for victims but also for their perpetrators although involvement in bullying is not a stable predictor of mastery of developmental tasks when childhood demographics, child intelligence, and psychopathology are taken into account.

Two decades after Olweus’ (1996) landmark study, there is little doubt that involvement in bullying victimization can jeopardize young people’s development. Studies have examined the health of victims (Copeland et al. 2013; Kretschmer et al. 2014; Ostrov and Kamper 2015) and individual variation in biomarkers such as inflammation (Copeland et al. 2014, Takizawa et al. 2015), and gene methylation (Ouellet-Morin et al. 2012). Being victimized also impedes on other areas in life such as financial status (Wolke et al. 2013), employment, and education (Takizawa et al. 2014) but prospective research into functional outcomes of bullying victimization has largely neglected the broad spectrum of developmental tasks young adults are required to master (cf. Havighurst 1948). In addition, comparably little is known about the perpetrators of bullying. Some studies reported higher externalizing problems (e.g., Bender and Lösel 2011) whereas others argued that bullying perpetration serves an evolutionarily adaptive function (Volk et al. 2012) which might explain why not all studies revealed negative correlates in perpetrators (Wolke et al. 2013). How bullies fare with respect to mastering developmental tasks has not been systematically studied.

Besides examining direct effects between bullying involvement and mastery of developmental tasks, we attend to recent calls for studies into potential buffers of consequences of bullying involvement by testing whether supportive relationships with parents diminish the risk for negative outcomes. Social support modulates associations between bullying victimization and internalizing maladjustment but very little is known as to whether supportive relationships with others also buffer negative effects on facets of normative development and whether the increased risk for bullying perpetrators can be attenuated by supportive relationships with close others.

## Developmental Tasks in Early Adulthood

Mastery of age-graded developmental tasks has been a central topic in developmental psychology for decades, with critical input from Havighurst (1948), who described various societal expectations for young adults, including the need to select a romantic partner, manage a home and rear children, find a social group, take on civic responsibility, get started in an occupation, and adjust to one’s masculine or feminine role. These tasks are progressive thus can only be tackled if tasks of childhood and adolescence have been mastered and, in turn, determine whether developmental tasks of middle and late adulthood are undertaken. In short, Havighurst (1948) suggested that mastery of early adult tasks is crucial for mid- and late adult development.

Similarly, Hutteman et al. (2014) argued that developmental tasks describe the establishment of age-dependent new roles (e.g., as romantic partner) and preparation for newly arising tasks (e.g., starting a family), which are closely related to personality development (Hutteman et al. 2014). Their update of Havighurst’s classification includes “continuing higher education” as task in early adulthood, which is in line with more recent theoretical accounts of developmental tasks (Nurmi 1993) that have pointed at the social and historical influence on the content of normative development.

Indeed, tasks and roles that were common for young adults a few decades ago are now deferred, with marriage and childbearing as prominent examples. According to official statistics, fewer than 20% of under-30-year-old Dutch adults are married and starting a family is postponed until the average age of 29 in Dutch women and 34 years in Dutch men (Latten 2004). These numbers are comparable to other Western countries (e.g., Bundeszentrale fuer politische Bildung 2016), thus some developmental tasks are now tackled remarkably later than suggested by Havighurst (1948). The greater diversity in early adulthood as a phase in life (Arnett 2000) is reflected in the varying content of contemporary conceptions of developmental tasks: For instance, academic attainment, social involvement and friendship, romantic relationships, work competence, and law-abiding conduct reflect “competent adaptive functioning” (*p.* 126, Roisman et al. 2004). Defining normative developmental tasks as “what many people […] commonly do, rather than what they should do” (*p*. 1221), Schulenberg et al. (2004) focus on educational attainment, work, financial autonomy, romantic involvement, peer involvement, substance abuse avoidance, and citizenship.

Life-course research in criminology offers another perspective on what it means to grow from an adolescent into an adult. In this field, this transition is characterized by desistance from behaviors that are perceived as relatively normative during adolescence such as delinquency and substance use. Moffitt (1993) argued that these behaviors serve as strategy to cope with the discrepancy between biological and social maturity. Once adolescents reach young adulthood, “proxy behaviors” are arguably not needed anymore and may actually harm someone’s status in adult contexts and further development. Consequently, norm compliance/law-abiding conduct (Barry et al. 2009; Roisman et al. 2004) and reduction or abstention from substance use (Barry et al. 2009; Schulenberg et al. 2004) have been discussed as additional competencies to be mastered in early adulthood.

In this study, we included tasks that have been deemed relevant by contemporary studies into the topic and largely follow Roisman et al.’s (2004) grouping into competence clusters. We study whether someone had established a romantic relationship as indicator of romantic/intimacy competence, their educational attainment and whether they are in work or continuing higher education as indicators of education/work competence, and financial and welfare payment independence as aspects of financial competence. In addition, we were interested in (near-) abstention from substances and law-abiding behavior.

## Developmental Tasks and Bullying Involvement

We propose that individual differences in mastery of developmental tasks are related to previous bullying involvement because peer relationships are of central importance in childhood and adolescence and crucial for future development. For instance, in line with attachment and social learning theories (Bandura and McClelland 1971; Connolly et al. 2000), positive peer experiences are associated with quality of interpersonal experiences later on and might be beneficial for healthy development overall (Viner et al. 2012) whereas negative peer experiences such as being victimized by peers are linked to psychological maladjustment (Copeland et al. 2014; Takizawa et al. 2014). It is feasible that links found for victim’s psychological adjustment also apply to other outcomes.

Although usually consciously initiated and sometimes discussed as evolutionarily adaptive, bullying perpetration has also been associated with problem outcomes including violence, substance use, delinquency, and antisocial personality disorder (Copeland et al. 2013; Farrington and Ttofi 2011). These outcomes reflect facets of general externalizing behavior of which bullying perpetration represents a developmentally appropriate symptom. As such, it should be linked to difficulties in a broader range of outcomes.

Offering first support for these assumptions, some studies have shown that bullying perpetrators and victims fare worse with respect to developmental tasks in the financial and education/work domains. Wolke et al. (2013) found significant associations between victimization and lower financial and educational status, and Takizawa et al. (2014) and Strøm et al. (2013) observed lower educational attainment and greater unemployment in adults who had been victimized in childhood or adolescence. Varhama and Björkqvist (2005) showed that unemployed individuals more often recalled victimization experiences and Brown and Taylor (2008) found that being bullied in childhood negatively affected earnings in adulthood. The latter study also examined outcomes for bullies and found lower educational attainment in late adolescence in this group (Brown and Taylor 2008).

Unfortunately, these studies did not examine mastery of developmental tasks in victims and perpetrators more broadly, thus it is unclear whether associations are as common as with measures of psychological adjustment. This lack of research not only results in incomplete knowledge on outcomes of bullying involvement, it also impedes systematic implementation of interventions that might reduce the negative effects of bullying involvement on development. Of course, it is possible that associations between bullying involvement and mastery of developmental tasks are explained by both constructs’ links to psychopathology. For instance, victimized adolescents might fail to master certain developmental tasks because of internalizing symptoms that may result from the victimization experience or may have been present already. Similarly, a perpetrator might continue to engage in crime in adulthood because of an underlying externalizing profile. To understand the relative contribution of bullying involvement to mastery of developmental tasks, such potential explanations need to be taken into account.

## Social Support as Buffer

Positive social relations are assumed to buffer against the negative sequelae of bullying-victimization (Ttofi et al. 2014) but the few studies on the topic focused on the classroom environment, such as teachers or classmates (Davidson and Demaray 2007) and peers (Holt and Espelage 2007; Rothon et al. 2011; Woods et al. 2009). Overall, isolated adolescents suffered greater stress symptoms after being victimized (Newman et al. 2005) whereas availability of social support reduced the effect of victimization on negative outcomes (Malecki and Demaray 2004). Turning to out-of-school contexts, Stadler et al. (2010)) showed that parental support protected against maladjustment in victimized girls. Rothon et al. (2011) reported that moderate levels of family support had a positive effect on victim’s educational achievement. In contrast, Holt and Espelage (2007) did not find a moderating role of maternal support on associations between bullying victimization and internalizing symptoms.

Aiming to consolidate these divergent findings and contributing to a still small number of studies that incorporate perpetrators of bullying, we concentrated on parental support as moderator on links between early adolescent victimization and perpetration and mastery of early adult developmental tasks. Although it is plausible that negative effects of victimization and perpetration can best be diminished if social support is available at time of exposure, we were particularly interested in support during the transition from adolescence through adulthood because of its temporal proximity to the outcomes under study. High levels of support are needed to finish school, find a job, and limit engagement in substance use, and those young adults who are supported in taking up new roles and develop adult competencies might suffer less from the consequences of earlier social insults. The role of parental support is less straightforward for perpetrators but if bullying is understood as a facet of a broader externalizing type, its developmental stability and link with early adult outcomes should also be diminished by particularly positive relationships with parents.

## Gender-Specific Patterns

Gender differences have been observed with respect to bullying perpetration, which, at least in its overt form, is more common in boys (Álvarez-García et al. 2015; Espelage 2014; Fekkes et al. 2005) and gender-specific associations have been established between bullying, its antecedents, and outcomes (Kretschmer et al. 2017; Sentse et al. 2015). Unfortunately, these studies did not examine associations between bullying involvement and mastery of normative developmental tasks, it is thus not clear to what extend boys and girls who were victims or perpetrators of bullying might differ with respect to mastering those tasks. We therefore examined gender-specificity in associations between bullying victimization and perpetration and mastery of early adult tasks and explored whether potential moderation effects by parental support depend on gender.

## Present Studies

Associations between bullying involvement in early adolescence and developmental tasks that are exemplary for mastering the transition to adulthood were examined in two contemporary longitudinal Dutch studies. Research on Adolescent Development and Relationships-Younger Cohort (RADAR-young) has followed individuals from early adolescence to early adulthood and TRacking Adolescents’ Individual Lives Survey (TRAILS) has followed individuals from pre-adolescence into their mid-twenties. These samples are similar with respect to general region and historical period but were collected in different parts of the country, thus links that are found in both samples provide support for generalizability at least to Dutch adolescents.

In both samples, we first examined whether early adolescent bullying perpetration and victimization were predictive of mastery of developmental tasks in early adulthood, specifically romantic relationship status as indicator of romantic competence, educational attainment (TRAILS only) and integration into the labor market or further education as indicators of education/work competence, welfare (in-) dependence and absence of financial problems as indicators of financial competence (TRAILS only), and law-abiding behavior and substance use as indicators of conduct. Overall, we expected individuals who have been involved in bullying as adolescents to fare worse with respect to mastering these developmental tasks.

Childhood demographics and child intelligence are important predictors of adult development and have served as control variables in other studies on outcomes of bullying involvement (Takizawa et al. 2014; Wolke et al. 2013), we thus estimated all models both unadjusted as well as adjusted for these potential confounders. Moreover, childhood psychopathology might be an important explanatory mechanisms for associations between bullying involvement and mastery of developmental tasks as bullying victimization and, to a lesser extent, perpetration are linked to psychopathology, concurrently and over time. In other words, those involved in bullying as victims or perpetrators are perhaps less likely to master developmental tasks because psychopathological symptoms prevent them from doing so. Thus, we also computed models in which internalizing and externalizing symptoms in childhood were added. We expected that confounders and childhood psychopathology reduce the strengths of associations between bullying involvement and mastery of developmental tasks.

Second, we examined potential moderating effects of parental support as buffer against the negative sequelae of bullying involvement. This question has hardly been researched, especially when outcomes refer to developmental tasks, but we tentatively expected that individuals who receive plenty of support while being confronted with early adult tasks would likely be less affected by previous bullying involvement.

Third, we explored whether boys and girls differed with respect to mastery of normative tasks following bullying involvement. Recent research conducted on the TRAILS sample showed that girls who bullied suffered greater maladjustment in adulthood whereas this effect was not observed for boys (Kretschmer et al. 2017). However, not all studies reported gender-specificity in associations between bullying involvement and later outcomes (cf. Turner et al. 2013), gender comparisons conducted in this study were thus of exploratory nature.

## Study 1: RADAR-Young

### Method

#### Participants and Procedure

RADAR-young is a longitudinal cohort study conducted in the central and western parts of the Netherlands. After obtaining ethical approval from the ethical committee of the University Medical Centre Utrecht, 429 randomly selected primary schools were approached of which 296 schools were willing to participate. For logistic reasons, data were collected in 230 schools. Families with students in the sixth grade who lived with two parents and at least one sibling aged 10 years or older were invited to participate if all family members had a firm grasp of the Dutch language. Written information about the study was distributed to family members and all individuals were required to provide informed consent prior to participation. Of 1081 approached families, 470 families refused participation after initial phone contact or during the first visit and 114 families failed to provide consent from all family members, resulting in a sample of 497 families who agreed to participate in the study. The vast majority of adolescents (95.2%) identified as Dutch, with the remainder identifying as Surinamese (1.4%) or another ethnicity including French, Australian, English, or Indonesian (3.4%). Parents were somewhat higher educated and less likely to work in elementary jobs than in the general population, accordingly, most (89%) families were classified as coming from medium or high socioeconomic status (SES), with a remaining 11% from families with low SES (Statistics Netherlands 1993).

Since the initial assessment in 2005, follow-ups have been conducted annually until early adulthood. Within each year of the study, trained research assistants visit families at home where participants complete questionnaires. This allows for research assistants to provide verbal instructions in addition to the written instructions that accompany the questionnaires. For the current study, data from three waves were used when adolescents were on average 13.0 (T1), 18.0 (T6), and 19.8 (T7) years old. Attrition analyses are described below.

#### Measures

Bullying victimization and perpetration were assessed at T1 using an adjusted and translated version of the Self-report of Aggression and Social Behavior Questionnaire (Morales and Crick 1998), a frequently used instrument (Linder et al. 2002; Murray-Close et al. 2010; Ostrov and Houston 2008) to assess interpersonal aggression, containing seven items assessing relational and physical aggression directed at the participant thus representing victimization (e.g., “Others tell mean rumors behind my back”) with response categories ranging from 1 = *completely untrue* to 7 = *completely true* and 16 items that refer to relational and physical aggression towards others thus representing perpetration (e.g., “If I am angry with someone, I try to exclude them from group activities”). The version used here was translated from English to Dutch by RADAR researchers using a forward/backward procedure but the translated version has not been independently validated. Scales showed sufficient reliability with Cronbach alpha’s of 0.78 and 0.76 for physical and relational victimization and 0.85 and 0.84 for bullying perpetration (physical and relational). Relational and physical subscales showed considerable overlap (*r =* 0.59 for victimization and *r* = 0.70 for bullying), we thus used averaged scores, *M* = 1.96, *SD* = 0.95 for bullying victimization and *M* = 1.72, *SD* = 0.76 for perpetration.

Developmental tasks were measured at T7 as follows: Romantic relationship status was referred to as having a boy- or a girlfriend. Education/work competence was conceptualized as being enrolled into a tertiary or other educational program or being in work (“Please indicate which educational program you follow” and “How many hours of paid work do you do per week?”). We distinguished between those young adults who were in work or enrolled in education from those who were neither. Moreover, we assessed law-abiding behavior, that is, whether someone had been in court, convicted or in contact with the police in the past year (“In the past 12 months, were you in contact with the police/did you have to appear in court/did you have to register with a probation officer?”), cannabis use in the past year, tobacco use (not regularly), and alcohol use (not more than three times per week). For reasons of consistency, we dichotomized all outcomes with 1 indicating mastery of a task (e.g., having a romantic partner, being in work or education, abstention from cannabis or near-abstention from tobacco use, low or moderate alcohol use) whereas 0 indicated that this task had not been mastered.

Social support was assessed at T6 using the eight-item Support subscale from the Network of Relationships Inventory (Furman and Buhrmester 1985), referring to mothers and fathers. Items included “Does this person admire and respect you?” and were assessed on a five-point scale ranging from 1 = *almost never/not at all* to 5 = *almost always/extremely much*. The inventory has been translated and used within the context of the Dutch CONAMORE study (De Goede et al. 2009; Selfhout et al. 2007). Cronbach’s alphas were 0.85 for maternal support (*M* = 3.60, *SD* = 0.64) and 0.88 for paternal support (*M* = 3.33, *SD* = 0.70). Because maternal and paternal support were strongly correlated (*r* = 0.55), we used a composite of both measures.

We calculated associations between bullying involvement and developmental tasks also while adjusting for childhood demographics (family SES, instability) and child intelligence: Family SES was constructed based on parents’ occupations at T1, with low SES referring to parents being unemployed or employed in low-status, elementary jobs. Mothers reported on their marital status, specifically their relationship to the biological father of the target participant and we dichotomized this information into married or cohabiting with biological father (85.4%) versus divorced from biological father of the child (family instability, 14.6%). Single parents were not included in RADAR-young as consequence of sampling requirements, thus stepfathers were present in the 14.6% of “instable families”. Child intelligence was measured using the Wechsler Intelligence Scale for Children-revised (vocabulary and block design subtests), the average on this scale was 102.1 (*SD* = 11.81).

Finally, we examined the role of child psychopathology and included the Reynolds Adolescent Depression Scale Total Score (Reynolds 2010; 23 items, 1 = *almost never* to 4 = *almost always*, Cronbach’s *α* = 0.93, *M* = 1.63, *SD* = 0.49) and Screen for Child Anxiety Related Emotional Disorders Total Score (SCARED, Birmaher et al. 1997; 38 items, 1 = *almost never* to 3 = *often*, Cronbach’s *α* = 0.91, *M* = 1.37, SD = 0.0.29). These scales have been translated into Dutch and back into English and back translations compared to original versions. Psychometric information has been published for the Dutch versions of the SCARED (Hale et al. 2005). Finally, we included the Youth Self Report Externalizing Scale (Achenbach 1991; 30 items, 0 = *never* to 2 = *often*, Cronbach’s *α* = 0.87, *M* = 0.35, *SD* = 0.24) based on a translation provided by Verhulst et al. (1997). All psychopathology assessments came from T1.

#### Attrition

Retention from T1 to T7 (77%) was more likely for participants from higher SES families, *t*(487) = 4.59, *p* < 0.001, where family stability was present, *t*(485) = 5.08, *p* < 0.001, and who had scored higher on the general intelligence test, *t*(444) = 2.35, *p* = 0.02. No differences with respect to childhood psychopathology, bullying involvement, or parental support were observed.

#### Analytic Strategy

Regression analyses were conducted in Mplus 7.4 (Muthén and Muthén 2012) using estimation procedures for categorical outcome variables and the full information maximum likelihood method, which makes uses of all available data points and avoids list- or pairwise deletion of cases with missing data. Victimization and perpetration models were computed separately, first unadjusted, thus entering only victimization or perpetration as predictor, in a second set of analyses adjusted for potential confounders (childhood demographics and intelligence), and, in a third set of analyses, with childhood psychopathology included as potential explanatory construct.

Following the main effect models, we tested moderating effects by parental support and gender by adding two-way interaction terms (victim x support; perpetrator x support, victim x gender; perpetrator x gender) in separate models. Finally, three-way interaction terms (victim x support x gender; perpetrator x support x gender) were added to explore whether the hypothesized role of parental support in diminishing negative effects of bullying involvement on mastery of developmental tasks varied for boys and girls. Note that all two-way interaction terms (e.g., victim x support, victim x gender, support x gender) were modeled in the three-way interaction models and that all models were computed while adjusting for childhood confounders and psychopathology, separately for bullying victimization and perpetration. Using Bonferroni-correction, we adjusted the significance threshold to the large number of interactions computed (12 per moderator), thus applied a *p*- threshold of 0.004 (0.05/12) to interpretation of interaction effects.

## Results

**Fig. 1:**
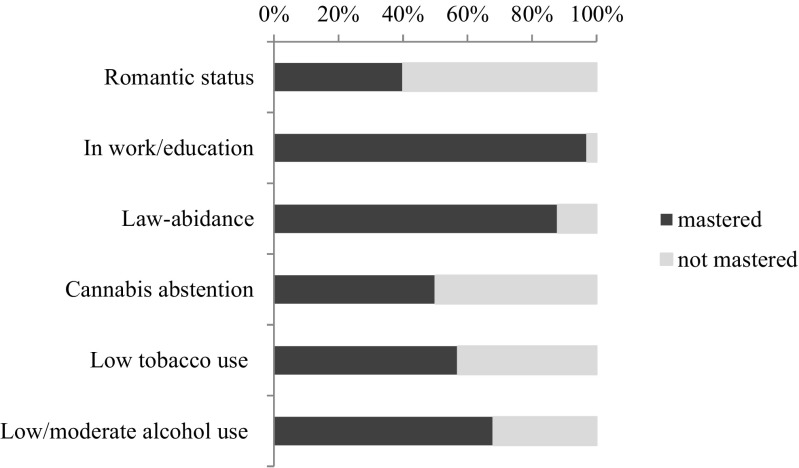
Mastery of developmental tasks in early adulthood (frequencies) in RADAR-young

Figure 1 depicts the distribution of participants who have mastered developmental tasks at age 19, independent of bullying involvement, indicating a balanced proportion for measures of romantic relationship status and substance use but an overweight of mastery of law-abidance and being enrolled in education or being employed. In other words, only few RADAR-young individuals were not enrolled in education or employed and were involved with crime.

Table 1 shows associations between bullying victimization and mastery of developmental tasks whereas associations for bullying perpetration are presented in Table 2, unadjusted in the upper part of the table, controlling for childhood confounders in the middle part, and with childhood internalizing and externalizing psychopathology added as predictors in the lower part. Both victims and perpetrators were less likely to abstain from tobacco use in early adulthood but this association was explained by externalizing problems, as was the association between bullying perpetration and decreased likelihood for law-abiding behavior (Table 2). Notably, a link between perpetration and cannabis use was detected when adjusting for childhood demographics, intelligence, and psychopathology but not in the unadjusted model.

**Table 1:** Associations between bullying victimization and mastery of developmental tasks (odds ratios) in RADAR-young

	Romantic status	In work/ education	Law-abidance	Cannabis abstention	Low tobacco use	Low/moderate alcohol use
Victimization (unadjusted)	1.02 (0.82/1.28)	1.15 (0.55/2.40)	0.78 (0.57/1.06)	0.91 (0.73/1.14)	**0.78 (0.62/0.98)**	0.99 (0.79/1.26)
Victimization	1.03 (0.82/1.28)	1.13 (0.52/2.46)	0.76 (0.56/1.05)	0.92 (0.74/1.16)	**0.77 (0.62/0.97)**	1.00 (0.79/1.27)
Family SES	1.30 (0.57/2.94)	**6.48 (1.56/26.89)**	0.46 (0.10/2.13)	1.06 (0.48/2.35)	**2.25 (1.01/5.04)**	0.63 (0.24/1.61)
Family instability	1.43 (0.73/2.81)	**0.24 (0.06/0.99)**	0.56 (0.23/1.39)	0.80 (0.41/1.59)	0.84 (0.42/1.66)	1.46 (0.67/3.20)
Child intelligence	1.00 (0.98/1.02)	1.04 (0.98/1.10)	1.02 (1.00/1.05)	**0.97 (0.96/0.99)**	1.01 (1.00/1.03)	**0.98 (0.96/1.00)**
Victimization	1.01 (0.76/1.34)	1.28 (0.47/3.49)	0.75 (0.49/1.14)	0.87 (0.65/1.16)	0.89 (0.67/1.20)	0.98 (0.72/1.32)
Family SES	1.30 (0.57/2.99)	**5.96 (1.37/25.93)**	0.47 (0.09/2.32)	1.17 (0.52/2.65)	2.19 (0.94/5.09)	0.66 (0.25/1.73)
Family instability	1.41 (0.72/2.78)	**0.21 (0.05/0.90)**	0.60 (0.24/1.53)	0.81 (0.41/1.62)	0.85 (0.42/1.70)	1.50 (0.68/3.32)
Child intelligence	1.00 (0.98/1.02)	1.04 (0.98/1.11)	1.02 (0.99/1.05)	**0.97 (0.95/0.99)**	1.02 (1.00/1.04)	**0.98 (0.96/1.00)**
Externalizing problems	1.27 (0.45/3.62)	1.09 (0.04/27.57)	**0.13 (0.03/0.57)**	0.57 (0.20/1.63)	**0.16 (0.05/0.48)**	0.58 (0.19/1.75)
Depressive symptoms	0.92 (0.47/1.79)	0.36 (0.05/2.78)	2.99 (0.99/8.99)	1.06 (0.55/2.07)	0.76 (0.38/1.54)	1.40 (0.69/2.86)
Anxiety	1.19 (0.42/3.39)	3.03 (0.11/82.37)	1.00 (0.19/5.37)	2.19 (0.76/6.32)	2.86 (0.92/8.85)	1.09 (0.35/3.35)

SES Socioeconomic status. Analyses are based on full information maximum likelihood logistic regression models. All outcomes were coded so that 0 = no mastery and 1 = mastery. Victimization and bullying were tested in separate models. Estimates are odds ratio and 95% confidence intervals are presented in brackets. Coefficients in bold font are statistically significant at *p* < 0.05

**Table 2:** Associations between bullying perpetration and mastery of developmental tasks (odds ratios) in RADAR-young

	Romantic status	In work/ education	Law-abidance	Cannabis abstention	Low tobacco use	Low/moderate alcohol use
Perpetration (unadjusted)	0.96 (0.73/1.26)	2.10 (0.60/7.41)	**0.68 (0.47/0.98)**	0.76 (0.58/1.00)	**0.63 (0.48/0.84)**	0.80 (0.60/1.06)
Perpetration	0.96 (0.73/1.27)	2.05 (0.60/6.78)	**0.69 (0.47/0.99)**	**0.74 (0.56/0.98)**	**0.63 (0.47/0.84)**	0.78 (0.59/1.04)
Family SES	1.29 (0.57/2.92)	**6** **.47 (1.53/27.27)**	0.45 (0.10/2.12)	1.06 (0.47/2.35)	**2.27 (1.01/5.13)**	0.61 (0.24/1.58)
Family instability	1.43 (0.73/2.81)	**0.23 (0.06/0.98)**	0.55 (0.22/1.37)	0.79 (0.40/1.57)	0.81 (0.41/1.62)	1.44 (0.66/3.16)
Child intelligence	1.00 (0.98/1.02)	1.04 (0.98/1.10	1.02 (0.99/1.05)	**0.97 (0.95/0.99)**	1.01 (0.99/1.03)	**0.98 (0.96/1.00)**
Perpetration	0.88 (0.61/1.27)	3.12 (0.72/13.65)	0.85 (0.52/1.39)	**0.67 (0.46/0.97)**	0.83 (0.57/1.21)	0.71 (0.48/1.03)
Family SES	1.35 (0.57/3.10)	**5.54 (1.27/24.15)**	0.44 (0.09/2.18)	1.21 (0.53/2.74)	2.19 (0.94/5.10)	0.69 (0.26/1.83)
Family instability	1.41 (0.72/2.77)	**0.21 (0.05/0.88)**	0.60 (0.24/1.54)	0.78 (0.39/1.57)	0.83 (0.41/1.68)	1.45 (0.66/3.20)
Child intelligence	1.00 (0.98/1.01)	1.05 (0.99/1.10)	1.02 (0.99/1.05)	**0.97 (0.95/0.99)**	1.02 (1.00/1.04)	**0.98 (0.96/1.00)**
Externalizing problems	1.65 (0.50/5.46)	0.28 (0.01/8.45)	**0.12 (0.02/0.65)**	1.01 (0.30/3.38)	**0.20 (0.06/0.69)**	1.08 (0.30/3.89)
Depressive symptoms	0.95 (0.49/1.83)	0.34 (0.05/2.59)	2.64 (0.90/7.71)	1.07 (0.55/2.06)	0.75 (0.38/1.50)	1.50 (0.74/3.05)
Anxiety	**1.17 (0.42/3.30)**	3.05 (0.11/82.30)	0.83 (0.16/4.32)	2.00 (0.70/5.73)	2.64 (0.87/8.05)	1.03 (0.34/3.17)

SES Socioeconomic status. Analyses are based on full information maximum likelihood logistic regression models. All outcomes were coded so that 0 = no mastery and 1 = mastery. Victimization and bullying were tested in separate models. Estimates are odds ratio and 95% confidence intervals are presented in brackets. Coefficients in bold font are statistically significant at *p* < 0.05

### Social Support and Gender as Moderators

Associations between bullying involvement and developmental tasks where re-estimated, now considering moderation by parental support and gender. While gender predicted cannabis abstention in both models (perpetration and victimization) directly, with girls being less likely to have abstained by the time of assessment, no moderation effects were found, neither in two- nor three-way interaction models (detailed results available upon request).

## Study 2: TRAILS

### Method

#### Procedure and Participants

The TRAILS sample was obtained in five municipalities in the north of the Netherlands, including urban and rural areas. Initial recruitment efforts targeted all children born between 1 October 1989 and 30 September 1990 (two municipalities) and 1 October 1990 and 30 September 1991 who attended a school. Thus, all 135 primary schools in the region were approached of which 122 agreed to participate. Parents and children in those schools were informed about the study goals and procedures through leaflets and contacted by a researcher who invited families to participate. Of the 3145 children reached through this procedure, 210 were excluded for various reasons such as severe mental retardation, physical illness or handicap, inability to participate, or limited mastery of the Dutch language (see Huisman et al. 2008) and 505 parents or children refused participation, resulting in a baseline sample of 2230 children (51% female). Both parents and children were asked to provide informed consent for participation. About a third of TRAILS participants came from families with low educated parents and 40% of parents worked in elementary jobs (Ganzeboom and Treiman 1996). Low income was more frequent than high income in this sample (30% versus 18%, middle income: 52%). The vast majority (90%) of participants were of Dutch ethnicity with only 230 adolescents growing up in families where one or both parents had been born outside the Netherlands, most commonly in Surinam, the Dutch Antilles, Indonesia, Morocco, and Turkey.

Since the initial assessment in 2001 (T1), when children were 11.1 years old, follow-ups have been conducted bi-or tri-annually with the most recent assessment (T6) conducted in 2016. The first three waves were largely school-based with incidental data collection done at the family home. The fourth and fifth wave were conducted using online questionnaires in addition to interviews and neuropsychological assessments taking place in central locations in the region and carried out by trained researchers. The sixth wave was conducted online, thus participants completed questionnaires at home. Ethical approval for the study was obtained from the Dutch national ethics committee CCMO and further details about the study have been published in several reports (Huisman et al. 2008; Nederhof et al. 2012; Oldehinkel et al. 2015; de Winter et al. 2005).

#### Measures

Bullying victimization and perpetration data were collected at T1 as part of broader instruments. That is, victimization was assessed using one item from the YSR (Achenbach 1991, “I am being bullied by others”), using the standard response format of 0 = *never*, 1 = *sometimes*, and 2 = *often*. The Dutch version of this questionnaire is based on Verhulst et al. (1997). Perpetration was assessed using one item from the Early Adolescent Temperament Questionnaire (Putnam et al. 2001, “I bully others without a reason”), using a five-point response format (1 = *almost never true* to 5 = *almost always true*). This instrument was translated using a forward/backward procedure at the University Medical Centre Groningen. Average scores were 0.38 (*SD* = 0.59) for victimization and 1.63 (*SD* = 0.95) for perpetration. 26.4% of participants were sometimes and 5.7% frequently victimized whereas 18.8% adolescents reported to bully others at least sometimes.

Developmental tasks were measured using self-reports at T5: Romantic relationship status as indicator of romantic competence was assessed by asking participants “Are you in a stable romantic relationship at this moment?”. Education/work competence was assessed as educational attainment (“What is the highest school diploma you have obtained?”) and education/work (“Are you currently following an educational program” and “Did you have paid work in the past month?”, see also Veldman et al. 2015, who studied this topic in TRAILS). Financial competence was assessed as financial problems (“In the past year, did you struggle financially?”, “Are you in debt with the bank or elsewhere?”) and welfare dependence (“Do you receive welfare payments?”). In addition, we asked whether someone had been in court, convicted or in contact with the police in the past year as indicators for law-abiding behavior, assessed cannabis use in the past year, occasional or frequently tobacco use, and alcohol use “more than three times a week”. As in Study 1, we coded all outcomes with a score of 1 indicating mastery of a task.

Social support was assessed at T4 using the Warmth subscale of the EMBU-C (Markus et al. 2003), which consists of four items (e.g., “My mum/dad tries to understand and help me when I am feeling sad”, 1 = *never* to 4 = *almost always*). This instrument is based on the Swedish Egna Minnen Beträffande Uppfostran (Perris et al. 1980) and the Dutch translation was developed taking into account the comprehensibility and interpretation of items by Dutch children (Markus et al. 2003). In the present sample, the scale was reliable with Cronbach’s *α* = 0.86 (mother) and 0.88 (father). Average scores were for mothers 3.31 (*SD* = 0.70) and for fathers 3.00 (*SD* = 0.85). Because maternal and paternal support were correlated (*r* = 0.62), a composite was used.

As in Study 1, we estimated associations between bullying involvement and mastery of developmental tasks also adjusted for childhood demographics and intelligence, and with psychopathology added to the model. Family SES was constructed from mothers’ and fathers’ educational and occupational levels and family income. Educational levels of parents were categorized in five categories based on the International Standard Classification of Occupations (Ganzeboom and Treiman 1996). Low family income was defined as a monthly net family income of less than €1135 per month, which amounted to a welfare payment at time of assessment. Family SES was measured as the average of the standardized five items (*α* = 0.84). Family instability contrasted participants who have lived with the same two parents since birth and those who have experienced either single parenthood since birth or some form of instability in the parental relationship (25.1%). Child intelligence was measured using the Wechsler Intelligence Scale for Children-revised (vocabulary and block design subtests, *M* = 97.19, *SD* = 15.0).

To examine the role of Child psychopathology as potential explanation for associations between bullying involvement and mastery of developmental tasks, three subscales from the Youth Self Report (Achenbach 1991; see Verhulst et al. 1997 for Dutch version) were employed that reflected a similar symptom spectrum as the instruments used in Study 1 all measured at T1 on a scale ranging from 0 = *not at all* to 2 = *often*, specifically depression/withdrawal (eight items, Cronbach’s *α* = 0.64, *M* = 0.34, *SD* = 0.29), anxiety (13 items, Cronbach’s *α* = 0.78, *M* = 0.33, *SD* = 0.27), and externalizing problems (32 items,^1^ Cronbach’s *α* = 0.86, *M* = 0.27, *SD* = 0.20).

#### Attrition

The T5 assessments used here were completed by 68% of the original sample, who differed from those that were lost to attrition in that they came from higher SES families, *t*(2186) = 12.41, *p* < 0.001, were family stability was present, *t*(2041) = 2.25, *p* = 0.02, who had scored higher on the general intelligence test, *t*(2219) = 11.92, *p* < 0.001, and less frequently reported bullying perpetration *t*(2044) = 2.52, *p* = 0.01. No differences were found for bullying victimization, child psychopathology, or parental support.

#### Analytic Strategy

As in Study 1, perpetration and victimization models were computed separately; unadjusted, adjusted for confounders (childhood demographics and intelligence), and with psychopathology entered as potentially explanatory construct. Following direct effect models, we examined parental support and gender as moderators, and, in three-way interaction models, tested whether parental support functioned as moderator differently for boys and girls. As in Study 1, two-way interactions were modeled in three-way interaction regressions, all models were computed separately for bullying victims and perpetrators, and corrected for multiple testing, resulting in a *p* - threshold of 0.003 (0.05/18).

## Results

### Developmental Tasks in Early Adulthood

**Fig. 2:**
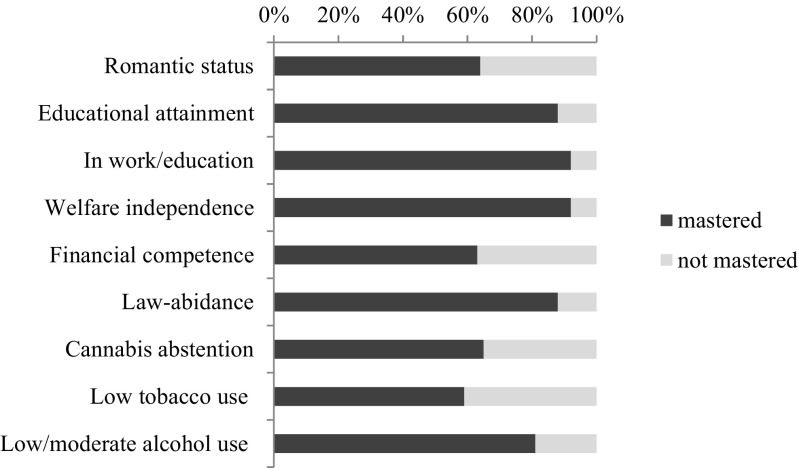
Mastery of developmental tasks in early adulthood (frequencies) in TRAILS

Figure 2 depicts the distribution of TRAILS participants who had mastered developmental tasks at time of assessment. All tasks had been mastered by more than half of the sample and only a minority of young adults was not in work or education, dependent on welfare, or had been involved with some form of criminal behavior.

Tables 3 and 4 present associations between bulling victimization, perpetration, and mastery of developmental tasks. In unadjusted models, victims were less likely to have mastered developmental tasks in the education/work and financial spectrum, but only mastery of welfare independence remained a significant outcome when adjusting for childhood confounders and taking child psychopathology into account.

**Table 3:** Associations between bullying-victimization mastery of developmental tasks (odds ratios) in TRAILS

	Romantic status	Educational attainment	In work/ education	Welfare independence	Financial competence	Law-abidance	Cannabis abstention	Low tobacco use	Low/moderate alcohol use
Victimization (unadjusted)	1.02 (0.84/1.25)	**0.73 (0.57/0.93)**	**0.59 (0.45/0.77)**	**0.49 (0.38/0.64)**	**0.82 (0.69//0.98)**	0.94 (0.72/1.23)	1.00 (0.83/1.20)	1.03 (0.86/1.23)	1.17 (0.93/1.48)
Victimization	0.99 0.81/1.21)	0.90 (0.69/1.18)	**0.70 (0.52/0.93)**	**0.59 (0.44/0.78)**	0.89 (0.74/1.06)	1.01 (0.77/1.31)	0.96 (0.79/1.16)	1.08 (0.90/1.29)	1.09 (0.86/1.37)
Family SES	**0.80 (0.67/0.94)**	**1.74 (1.37/2.12)**	**1.72 (1.29/2.28)**	**1.56 (1.17/2.07)**	1.11 (0.95/1.30)	1.08 (0.86/1.36)	**0.72 (0.62/0.85)**	1.12 (0.97/1.30)	**0.75 (0.62/0.91)**
Family instability	**0.74 (0.56/0.99)**	**0.61 (0.42/0.87)**	**0.62 (0.41/0.94)**	**0.40 (0.27/0.61)**	**0.46 (0.35/0.60)**	**0.57 (0.40/0.82)**	**0.53 (0.40/0.69)**	**0.77 (0.59/0.99)**	1.11 (0.79/1.58)
Child intelligence	1.00 (0.99/1.01)	**1.04 (1.03/1.05)**	**1.02 (1.01/1.04)**	**1.04 (1.02/1.05)**	1.01 (1.00/1.01)	1.01 (1.00/1.02)	**0.98 (0.98/0.99)**	1.00 (1.00/1.01)	**0.99 (0.98/1.00)**
Victimization	1.03 (0.83/1.29)	0.90 (0.68/1.21)	0.76 (0.55/1.04)	**0.65 (0.47/0.89)**	1.05 (0.86/1.29)	1.04 (0.77/1.42)	0.95 (0.77/1.17)	1.14 (0.93/1.39)	1.05 (0.82/1.36)
Family SES	**0.80 (0.67/0.94)**	**1.73 (1.36/2.20)**	**1.72 (1.30/2.29)**	**1.56 (1.17/2.08)**	1.12 (0.95/1.31)	1.06 (0.84/1.34)	**0.71 (0.60/0.83)**	1.11 (0.95/1.29)	**0.75 (0.62/0.90)**
Family instability	**0.75 (0.56/0.99)**	**0.61 (0.43/0.88)**	**0.64 (0.42/0.97)**	**0.41 (0.27/0.61)**	**0.46 (0.35/0.60)**	**0.56 (0.39/0.82)**	**0.53 (0.40/0.69)**	0.77 (0.59/1.00)	1.11 (0.78/1.57)
Child intelligence	1.00 (0.99/1.01)	**1.04 (1.03/1.05)**	**1.02 (1.01/1.04)**	**1.04 (1.02/1.05)**	1.01 (1.00/1.01)	1.01 (1.00/1.02)	**0.98 (0.98/0.99)**	1.01 (1.00/1.01)	**0.99 (0.98/1.00)**
Depression	1.08 (0.61/1.89)	0.66 (0.31/1.43)	**0.30 (0.13/0.71)**	0.51 (0.21/1.21)	0.75 (0.45/1.25)	2.12 (0.96/4.70)	1.54 (0.90/2.62)	0.97 (0.59/1.60)	1.81 (0.95/3.45)
Anxiety	0.89 (0.50/1.59)	**2.36 (1.00/5.54)**	**2.76 (1.06/7.18)**	1.19 (0.47/3.02)	0.84 (0.49/1.44)	2.14 (0.93/4.96)	1.31 (0.75/2.27)	**1.84 (1.08/3.13)**	0.97 (0.50/1.89)
Externalizing problems	0.66 (0.33/1.33)	0.48 (0.18/1.24)	0.66 (0.23/1.94)	0.88 (0.29/2.64)	**0.35 (0.18/0.67)**	**0.05 (0.02/0.12)**	**0.24 (0.12/0.46)**	**0.17 (0.09/0.33)**	**0.41 (0.19/0.90)**

SES Socioeconomic status. Analyses are based on full information maximum likelihood logistic regression models. All outcomes were coded so that 0 = no mastery and 1 = mastery. Victimization and bullying were tested in separate models. Estimates are odds ratio and 95% confidence intervals are presented in brackets. Coefficients in bold font are statistically significant at *p* < 0.05

**Table 4:** Associations between bullying perpetration and mastery of developmental tasks (odds ratios) in TRAILS

	Romantic status	Educational attainment	In work/ education	Welfare independence	Financial competence	Law-abidance	Cannabis abstention	Low tobacco use	Low/moderate alcohol use
Perpetration (unadjusted)	0.96 (0.84/1.09)	0.87 (0.74/1.03)	**0.74 (0.62/0.91)**	**0.78 (0.64/0.94)**	0.94 (0.83/1.06)	**0.70 (0.60/0.82)**	0.89 (0.79/1.01)	**0.81 (0.71/0.91)**	0.96 (0.82/1.10)
Perpetration	0.93 (0.82/1.07)	1.00 (0.84/1.19)	0.83 (0.69/1.01)	0.88 (0.72/1.07)	0.96 (0.84/1.08)	**0.72 (0.61/0.85)**	**0.84 (0.74/0.96)**	**0.82 (0.73/0.93)**	0.91 (0.78/1.06)
Family SES	**0.79 (0.67/0.93)**	**1.77 (1.39/2.24)**	**1.74 (1.31/2.30)**	**1.63 (1.23/2.16)**	1.12 (0.96/1.31)	1.03 (0.82/1.29)	**0.71 (0.61/0.83)**	1.09 (0.93/1.26)	**0.74 (0.61/0.89)**
Family instability	**0.74 (0.56/0.98)**	**0.60 (0.42/0.86)**	**0.60 (0.40/0.91)**	**0.39 (0.26/0.59)**	**0.45 (0.35/0.59)**	**0.57 (0.39/0.81)**	**0.52 (0.40/0.67)**	**0.77 (0.59/0.99)**	1.12 (0.79/1.59)
Child intelligence	1.00 (0.99/1.01)	**1.04 (1.03/1.05)**	**1.02 (1.01/1.04)**	**1.04 (1.02/1.05)**	1.00 (1.00/1.01)	1.01 (1.00/1.02)	**0.98 (0.98/0.99)**	1.00 (1.00/1.01)	**0.99 (0.98/1.00)**
Perpetration	0.96 (0.83/1.11)	1.05 (0.87/1.28)	0.85 (0.69/1.05)	0.91 (0.73/1.21)	1.07 (0.93/1.23)	**0.83 (0.70/1.00)**	0.92 (0.80/1.05)	0.92 (0.80/1.05)	0.94 (0.79/1.11)
Family SES	**0.79 (0.67/0.93)**	**1.77 (1.39/2.25)**	**1.73 (1.30/2.29)**	**1.63 (1.22/2.16)**	1.12 (0.96/1.31)	1.03 (0.82/1.31)	**0.71 (0.60/0.83)**	1.08 (0.93/1.26)	**0.74 (0.61/0.89)**
Family instability	**0.75 (0.56/0.99)**	**0.61 (0.43/0.87)**	**0.62 (0.41/0.94)**	**0.40 (0.27/0.60)**	**0.46 (0.36/0.60)**	**0.56 (0.38/0.80)**	**0.52 (0.40/0.69)**	0.78 (0.60/1.01)	1.11 (0.78/1.57)
Child intelligence	1.00 (0.99/1.01)	**1.04 (1.03/1.05)**	**1.02 (1.01/1.04)**	**1.04 (1.02/1.05)**	1.01 (1.00/1.01)	1.01 (1.00/1.02)	**0.98 (0.98/0.99)**	1.00 (1.00/1.01)	**0.99 (0.98/1.00)**
Depression	1.08 (0.62/1.89)	0.64 (0.30/1.37)	**0.26 (0.11/0.59)**	**0.42 (0.18/0.99)**	0.77 (0.46/1.28)	2.14 (0.97/4.69)	1.50 (0.89/2.53)	1.02 (0.62/1.67)	1.85 (0.98/3.49)
Anxiety	0.90 (0.51/1.60)	2.26 (0.97/5.26)	2.45 (0.95/6.34)	0.97 (0.39/2.46)	0.86 (0.51/1.47)	2.17 (0.94/4.98)	1.27 (0.74/2.19)	**1.92 (1.14/3.25)**	0.99 (0.52/1.90)
Externalizing problems	0.71 (0.34/1.49)	0.43 (0.16/1.16)	0.83 (0.27/2.62)	0.94 (0.29/3.01)	**0.31 (0.15/0.62)**	**0.07 (0.03/0.17)**	**0.27 (0.13/0.55)**	**0.21 (0.10/0.41)**	0.47 (0.20/1.09)

SES Socioeconomic status. Analyses are based on full information maximum likelihood logistic regression models. All outcomes were coded so that 0 = no mastery and 1 = mastery. Victimization and bullying were tested in separate models. Estimates are odds ratio and 95% confidence intervals are presented in brackets. Coefficients in bold font are statistically significant at *p* < 0.05

Bullying perpetrators showed decreased odds for being in work/education and independent of welfare, and were also less likely to live law-abiding lives and abstain from tobacco use. While associations with tobacco use were retained when we controlled for childhood demographics and intelligence, externalizing problems explained most associations between bullying perpetration and developmental tasks, as evident from results presented in the lower part of Table 4. Solely a decreased likelihood to live a law-abiding life was predicted by bullying perpetration, over and above childhood confounders and child psychopathology. Perpetration was again linked to cannabis use though only in the model controlling for childhood confounders but not in the unadjusted model or when associations between psychopathology and cannabis use were estimated as well.

### Social Support and Gender as Moderators

Following main effect estimations, we computed models in which interactions between bullying perpetration or victimization and social support by mothers and fathers, and gender, respectively, were included (victim x support; perpetrator x support, victim x gender; perpetrator x gender). Direct effects of parental support and gender were detected, and although estimates varied slightly depending on whether bullying perpetration or victimization were part of the model, the overall pattern of results was largely the same. Specifically, higher levels of parental support predicted being in education or work, social welfare independence, financial competence, cannabis abstention, and low/moderate alcohol use, as well as law-abiding conduct in the bullying perpetration model. Gender was associated with educational attainment, law-abiding behavior, cannabis abstention and low to moderate (instead of heavy) alcohol use; in all cases were boys less likely to have mastered the developmental tasks.

As in Study 1, none of the interactions involving parental support or gender achieved statistical significance, neither in two- nor three-way models. Thus, associations between bullying involvement in early adolescence and mastery of developmental tasks did not vary as a function of either parental support or gender.

## Discussion

Given the prominent role of peers in adolescence, it is hardly surprising that bullying involvement can have a detrimental impact on psychological health (Bender and Lösel 2011; Ttofi et al. 2011) although research on perpetrators is less conclusive than that on victims (Wolke et al. 2013). Of course, positive development and wellbeing are not only dependent on avoiding psychological illness but characterized also by successful accomplishment of various developmental tasks, which were the focus of the current study.

We examined whether bullying involvement in early adolescence was linked to mastery of developmental tasks in early adulthood in two contemporary Dutch cohorts – RADAR-young, where participants were just under 20 years old at time of assessment, and TRAILS, where participants were approximately 22 years old. Overall, similar rates of mastery were observed for law-abidance and tobacco use in both cohorts whereas romantic relationships were more common amongst TRAILS participants, and alcohol and cannabis use were more common amongst RADAR-young participants, probably a function of the slightly different ages of participants.

Associations of victimization with education/work and financial competence were detected in the TRAILS sample, though only the link between victimization and welfare dependence remained statistically significant after controlling for a range of potential confounders and when associations between child psychopathology and developmental tasks were estimated simultaneously. In RADAR-young, victimized teens were at greater risk to smoke at least occasionally by early adulthood though this link weakened to non-significance when adjusted for childhood demographics, intelligence, and psychopathology.

Overall, these results indicate that victims of bullying fare worse not only with respect to mental health as shown in previous research but to some extent also display greater difficulties in mastering developmental tasks. It is important to note, however, that associations were fewer and much less systematic than expected, certainly not as common as for mental health outcomes. Moreover, adjusting for childhood demographics and psychopathology reduced effect sizes, partly considerably, which suggests that in light of various other childhood risks, bullying victimization is less crucial for mastery of early adult developmental tasks.

Of particular interest to this study given the relative lack and ambiguity of knowledge concerning its outcomes, bullying perpetration was initially linked to greater substance use and less law-abiding behavior in both samples, though these associations were mostly explained by externalizing symptoms. Only the reduced likelihood for a law-abiding live in bullying perpetrators was retained in TRAILS, tentatively suggesting that even in light of various childhood risks for crime involvement, bullying perpetration increased the risk for an antisocial lifestyle (Hussong et al. 2004). Programs focused on helping victims deal with their plight should thus also keep an eye on the development of the perpetrators.

Curiously, in both samples bullying perpetration was associated with decreased likelihood for cannabis abstention, though only when childhood demographics and intelligence were part of the model. What is more, cannabis abstention was less likely in young adults from well-off, stable family backgrounds who had scored higher on child intelligence tests. These measures thus appear to function as suppressors but the changes in effect sizes are negligible.

Turning to buffering effects of social support, we hypothesized that the mastery of developmental tasks should be facilitated if parents are supportive, even if prior bullying victimization or perpetration might reduce the likelihood of a smooth transition into adulthood. Indeed, parental support was associated with mastery of developmental tasks (at least in TRAILS), but no interactions between parental support and bullying victimization were observed. We purposely focused on parental support in late adolescence but it may be that supportive social relationships need to be experienced more temporally proximal to the victimization experience to really buffer their impact. It is difficult to imagine the form of support that perpetrators need at the time that they bully others but parents may be able to set off negative effects by offering strategies to change this behavior early on. Either way, links between bullying involvement and mastery of developmental tasks were modest, there is thus little effect that needs to be buffered.

Finally, we explored whether associations between bullying victimization and perpetration and mastery of early adult tasks differed between boys and girls. In contrast to studies focusing on psychological adjustment, we did not find evidence for gender specificity in associations. This also applied to three-way interaction models were we examined whether the role of parental support in buffering or elevating effects of bullying victimization and perpetration on early adult tasks showed gender specificity. For the time being, we conclude that involvement in bullying is associated with mastery of developmental tasks to the same (partial) extend for boys and girls and that parental support makes little difference for either of them but, given divergent findings in past research, future research into correlates of perpetration and victimization is still advised to explore whether gender or social support might modulate associations.

Though not a focus of the study, our results confirm important roles for childhood risks. Family SES in early adolescence predicted whether or not young adults in both RADAR-young and TRAILS participated in the labor market or tertiary or continuing education and, at least in TRAILS, family instability contributed to risk for low educational attainment, lack of financial competence, and welfare dependence. These were anticipated associations, yet, the impact of these childhood demographics on mastery of developmental tasks almost a decade later is still striking, particularly considering that these links remained largely stable when child psychopathology was added to the models. Externalizing problems in early adolescence stably predicted crime involvement and substance use in early adulthood whereas associations between developmental tasks and internalizing problems were more scattered and not consistent across samples.

Finally, in TRAILS, higher intelligence and higher SES in early adolescence increased the likelihood for alcohol and cannabis use in early adulthood, the link between intelligence and alcohol use was also detected in RADAR-young. It is likely that these substances tend to be used among students and that university attendance is more likely in high-SES and high-intelligence individuals. Explicitly testing this explanation went beyond the scope of the current study but the pattern of associations underlines the complex interplay of risks and protective factors in young people’s mastery of developmental tasks.

### Implications of Findings

The results of this study clearly show that long-term correlates of bullying perpetration and victimization are less common and stable when focusing on developmental task, especially compared to research on psychological outcomes. Initial associations were reduced when childhood demographics, IQ, and psychopathology were taken into account, suggesting that whether or not a young adult successfully masters developmental milestones is better predicted by family circumstances than by experiences with peers in early adolescence. Prospective and comprehensive studies that focus on the pathways explaining why young people growing up in low SES families and fluctuation in terms of parenting figures are at greater risk will be informative in further elucidating underlying pathways. This work could inform practical approaches to improving young people’s chances to successfully master the transition to adulthood.

### Limitations and Future Directions

Despite the strengths of this study including the availability of two cohorts and data spanning almost a decade between bullying involvement and mastery of developmental tasks, a number of limitations need to be considered. Firstly, self-reports of bullying involvement were used and consisted of only one item each in TRAILS. This is not an optimal way to assess bullying and increases the chance for shared method variance. In TRAILS, peer nominations of bullying and victimization were available but many associations showed extremely large confidence intervals, thus results appeared less reliable. It is, of course, possible that associations are due to a confounding self-perception of victims, but in our opinion, these associations are central and most important if they contribute to maladjustment and failure in mastering developmental tasks.

Second, RADAR-young and TRAILS youth live in the Netherlands, were assessed at approximately the same time and with respect to similar life domains, which makes these cohorts overall very similar and comparable. However, TRAILS participants were approximately three years older than RADAR-young participants, which probably influenced our results as mastery of some tasks is more normative at 22 than at 19, whereas failure to do so is more indicative of problem development at 22 than at 19. Moreover, the TRAILS cohort comprises of more than four times as many individuals as RADAR-young and was thus much better suited to detect small effects.

Third, we conceptualized mastery of developmental tasks according to previous research and plausibility. For some outcomes, this resulted in low prevalence rates. We chose this approach in line with other studies into outcomes of bullying victimization that examined psychiatric diagnoses with similarly low prevalence rates but realize that this strategy was not always reflected in the ratio of mastery/non-mastery of a task. The imbalance of mastery versus non-mastery ratio between outcomes might have influenced the likelihood for obtaining statistically significant results.

Fourth, other factors might be of explanatory importance for mastering developmental tasks, including adolescents’ mental health (Veldman et al. 2014), and social relationships experienced between pre-adolescence and early adulthood. For instance, other peer difficulties such as social alienation and negative life events but also macro-contextual factors can influence the ease with which young adults master developmental tasks, specifically economically related ones. That is, participants of RADAR-young and TRAILS were in late adolescence, finishing secondary school, and deciding on finding a job or going to university when the economic crisis hit the Netherlands in the late 2000s. It is likely that such historical changes affect whether young people successfully master the transition into adulthood – irrespective of social relationships. In other words, whereas bullying can play a prominent role in affecting future adjustment when other risks are absent, the relative contribution of it might be a lot less important in times of social change.

Fifth, intimacy competence, being good at romantic relationships, and learning to live with a partner are conceptualizations of the task to enter into a romantic relationship in other studies. We examined whether or not someone had a boy- or girlfriend and found no associations with bullying victimization or perpetration. We could have conceptualized intimacy differently, for instance by using the NRI Intimacy scale for partners of RADAR-young participants. Completing this scale was preempted by relationship status and those who did indicate to have a partner scored on average 3.9 on a scale from 1 to 5, with a lowest score of 2.4. In other words, practically everyone in RADAR-young who was in a relationship also experienced high levels of intimacy with their partner. The same pattern was observed for TRAILS, where those in a relationships completed the Investment Model Scale (Rusbult et al. 1998). As in RADAR-young, scores for relationship satisfaction clustered at the positive end, we thus have confidence that our comparably crude assessment of romantic competence reflects positive experiences quite well while staying consistent with the dichotomous conceptualization of other outcomes.

Sixth, theoretical accounts of developmental tasks and other empirical studies include concepts of citizenship, referring to charity donations or engagement in voluntary work (Schulenberg et al. 2004), or assess civic responsibility as facet of work competence (Hutteman et al. 2014). Others refer additionally to personal values and emotional independence from parents (e.g. Barry et al. 2009). Unfortunately, we did not have such measures available in one or both cohorts, thus could not test such whether this competence was mastered less well by victims or perpetrators of bullying.

### Conclusion

Based on two contemporary Dutch cohorts, we examined whether bullying perpetration and victimization in early adolescence affected mastery of normative developmental tasks in romantic, education, work, financial, and conduct domains in early adulthood. In both cohorts, perpetrators of bullying were more likely to use substances and less likely to lead a law-abiding life. Findings varied for victims - in TRAILS they were less likely to be in education or work and less financially competent, while in RADAR-young, they were more likely smoke at least occasionally. The strengths of associations was reduced when adjusted for other childhood risks and when childhood psychopathology was examined as potential explanation for links between bullying involvement and mastery of developmental tasks, which demonstrates that, relative to other childhood risks, bullying involvement is less strongly implied in mastery of early adult developmental tasks.

## Footnotes

1 Slightly different selections of externalizing items from the complete YSR were used in RADAR-young and TRAILS, the majority of items (26) are identical.

## References

[CR1] Achenbach TM (1991). Manual for the youth self-report and 1991 profile.

[CR2] Álvarez-García D, García T, Núñez JC (2015). Predictors of school bullying perpetration in adolescence: A systematic review. Aggression and Violent Behavior.

[CR3] Arnett JJ (2000). Emerging adulthood: A theory of development from the late teens through the twenties. American Psychologist.

[CR4] Bandura A, McClelland DC (1971). Social learning theory.

[CR5] Barry CM, Madsen SD, Nelson LJ, Carroll JS, Badger S (2009). Friendship and romantic relationship qualities in Emerging adulthood: Differential associations with identity development and achieved adulthood criteria. Journal of Adult Development.

[CR6] Bender D, Lösel F (2011). Bullying at school as a predictor of delinquency, violence and other anti-social behaviour in adulthood. Criminal Behaviour and Mental Health.

[CR7] Birmaher B, Khetarpal S, Brent D, Cully M, Balach L, Kaufman J, Neer SM (1997). The screen for child anxiety related emotional disorders (SCARED): Scale construction and psychometric characteristics. Journal of the American Academy of Child and Adolescent Psychiatry.

[CR8] Brown S, Taylor K (2008). Bullying, education and earnings: Evidence from the National Child Development Study. Economics of Education Review.

[CR9] Bundeszentrale fuer politische Bildung (2016). Datenreport 2016 - Ein Sozialbericht für die Bundesrepublik Deutschland.

[CR10] Connolly J, Furman W, Konarski R (2000). The role of peers in the emergence of heterosexual romantic relationships in adolescence. Child Development.

[CR11] Copeland WE, Wolke D, Angold A, Costello EJ (2013). Adult psychiatric outcomes of bullying and being bullied by peers in childhood and adolescence. JAMA Psychiatry.

[CR12] Copeland WE, Wolke D, Lereya ST, Shanahan L, Worthman C, Costello EJ (2014). Childhood bullying involvement predicts low-grade systemic inflammation into adulthood. Proceedings of the National Academy of Sciences.

[CR13] Davidson LM, Demaray MK (2007). Social support as a moderator between victimization and internalizing-externalizing distress from bullying. School Psychology Review.

[CR14] De Goede IH, Branje SJ, Delsing MJ, Meeus WH (2009). Linkages over time between adolescents’ relationships with parents and friends. Journal of Youth and Adolescence.

[CR15] de Winter AF, Oldehinkel AJ, Veenstra R, Brunnekreef JA, Verhulst FC, Ormel J (2005). Evaluation of non-response bias in mental health determinants and outcomes in a large sample of pre-adolescents. European Journal of Epidemiology.

[CR16] Espelage DL (2014). Ecological theory: Preventing youth bullying, aggression, and victimization. Theory into Practice.

[CR17] Farrington DP, Ttofi MM (2011). Bullying as a predictor of offending, violence and later life outcomes. Criminal Behaviour and Mental Health.

[CR18] Fekkes M, Pijpers FI, Verloove-Vanhorick SP (2005). Bullying: Who does what, when and where? Involvement of children, teachers and parents in bullying behavior. Health Education Research.

[CR19] Furman W, Buhrmester D (1985). Children’s perceptions of the personal relationships in their social networks. Developmental Psychology.

[CR20] Ganzeboom HB, Treiman DJ (1996). Internationally comparable measures of occupational status for the 1988 International standard classification of occupations. Social Science Research.

[CR21] Hale WW, Raaijmakers Q, Muris P, Meeus W (2005). Psychometric properties of the screen for child anxiety related emotional disorders (SCARED) in the general adolescent population. Journal of the American Academy of Child and Adolescent Psychiatry.

[CR22] Havighurst RJ (1948). Developmental tasks and education.

[CR23] Holt MK, Espelage DL (2007). Perceived social support among bullies, victims, and bully-victims. Journal of Youth and Adolescence.

[CR24] Huisman M, Oldehinkel AJ, Winter AD, Minderaa RB, Bildt AD, Huizink AC (2008). Cohort profile: The Dutch “TRacking adolescents” individual lives’ survey’; TRAILS. International Journal of Epidemiology.

[CR25] Hussong AM, Curran PJ, Moffitt TE, Caspi A, Carrig MM (2004). Substance abuse hinders desistance in young adults’ antisocial behavior. Development and Psychopathology.

[CR26] Hutteman R, Hennecke M, Orth U, Reitz AK, Specht J (2014). Developmental tasks as a framework to study personality development in adulthood and old age. European Journal of Personality.

[CR27] Kretschmer T, Barker ED, Dijkstra JK, Oldehinkel AJ, Veenstra R (2014). Multifinality of peer victimization: Maladjustment patterns and transitions from early to mid-adolescence. European Child & Adolescent Psychiatry.

[CR28] Kretschmer, T., Veenstra, R., Deković, M., & Oldehinkel, A. J. (2017). Bullying development across adolescence, its antecedents, outcomes, and gender-specific patterns. *Development and Psychopathology*. Advance Online Publication. doi:10.1017/S0954579416000596. 10.1017/S0954579416000596 27417540

[CR29] Latten J (2004). Trends in samenwonen en trouwen.

[CR30] Linder JR, Crick NR, Collins WA (2002). Relational aggression and victimization in young adults’ romantic relationships: Associations with perceptions of parent, peer, and romantic relationship quality. Social Development.

[CR31] Malecki CK, Demaray MK, Espelage DL, Swearer SM (2004). The role of social support in the lives of bullies, victims, and bully-victims. Bullying in American schools: A social-ecological perspective on prevention and intervention.

[CR32] Markus MT, Lindhout IE, Boer F, Hoogendijk TH, Arrindell WA (2003). Factors of perceived parental rearing styles: The EMBU-C examined in a sample of Dutch primary school children. Personality and Individual Differences.

[CR33] Moffitt TE (1993). Adolescence-limited and life-course-persistent antisocial behavior: A developmental taxonomy. Psychological Review.

[CR34] Morales J, Crick N (1998). *Self-report measure of aggression and victimization.* Unpublished measure.

[CR35] Murray-Close D, Ostrov JM, Nelson DA, Crick NR, Coccaro EF (2010). Proactive, reactive, and romantic relational aggression in adulthood: Measurement, predictive validity, gender differences, and association with intermittent explosive disorder. Journal of Psychiatric Research.

[CR36] Muthén LK, Muthén BO (2012). Mplus User’s Guide.

[CR37] Nederhof E, Jorg F, Raven D, Veenstra R, Verhulst FC, Ormel J, Oldehinkel AJ (2012). Benefits of extensive recruitment effort persist during follow-ups and are consistent across age group and survey method. The TRAILS study. BMC Medical Research Methodology.

[CR38] Newman ML, Holden GW, Delville Y (2005). Isolation and the stress of being bullied. Journal of Adolescence.

[CR39] Nurmi J-E (1993). Adolescent development in an age-graded context: The role of personal beliefs, goals, and strategies in the tackling of developmental tasks and standards. International Journal of Behavioral Development.

[CR40] Oldehinkel AJ, Rosmalen JG, Buitelaar JK, Hoek HW, Ormel J, Raven D (2015). Cohort Profile Update: The TRacking Adolescents’ Individual Lives Survey (TRAILS). International Journal of Epidemiology.

[CR41] Olweus D (1996). Bullying at school: Knowledge Base and an effective intervention Programa. Annals of the new York Academy of Sciences.

[CR42] Ostrov JM, Houston RJ (2008). The utility of forms and functions of aggression in Emerging adulthood: Association with personality disorder symptomatology. Journal of Youth and Adolescence.

[CR43] Ostrov JM, Kamper KE (2015). Future directions for research on the development of relational and physical peer victimization. Journal of Clinical Child & Adolescent Psychology.

[CR44] Ouellet-Morin I, Wong CC, Danese A, Pariante CM, Papadopoulos AS, Mill J, Arseneault L (2012). Increased serotonin transporter gene (SERT) DNA methylation is associated with bullying victimization and blunted cortisol response to stress in childhood: A longitudinal study of discordant monozygotic twins. Psychological Medicine.

[CR45] Perris C, Jacobsson L, Lindström H, von Knorring L, Perris H (1980). Development of a new inventory for assessing memories of parental rearing behaviour. Acta Psychiatrica Scandinavica.

[CR46] Putnam, S. P., Ellis, L. K., & Rothbart, M. K. (2001). The structure of temperament from infancy through adolescence. In A. Eliasz &, Angleitner (Eds.) *Advances in Research on Temperament* (pp. 165—182). Lengerich: Pabst Scientist Publisher.

[CR47] Reynolds WM (2010). Reynolds adolescent depression scale.

[CR48] Roisman GI, Masten AS, Coatsworth JD, Tellegen A (2004). Salient and Emerging developmental tasks in the transition to adulthood. Child Development.

[CR49] Rothon C, Head J, Klineberg E, Stansfeld S (2011). Can social support protect bullied adolescents from adverse outcomes? A prospective study on the effects of bullying on the educational achievement and mental health of adolescents at secondary schools in East London. Journal of Adolescence.

[CR50] Rusbult CE, Martz JM, Agnew CR (1998). The investment model scale: Measuring commitment level, satisfaction level, quality of alternatives, and investment size. Personal Relationships.

[CR51] Schulenberg JE, Bryant AL, O’Malley PM (2004). Taking hold of some kind of life: How developmental tasks relate to trajectories of well-being during the transition to adulthood. Development and Psychopathology.

[CR52] Selfhout MHW, Branje SJT, Meeus WHJ (2007). Similarity in adolescent best friendships: The role of gender. Netherlands Journal of Psychology.

[CR53] Sentse M, Kretschmer T, Salmivalli C (2015). The longitudinal interplay between bullying, victimization, and social status: Age-related and gender differences. Social Development.

[CR54] Stadler C, Feifel J, Rohrmann S, Vermeiren R, Poustka F (2010). Peer-victimization and mental health problems in adolescents: Are parental and school support protective?. Child Psychiatry and Human Development.

[CR55] Statistics Netherlands (1993). Standaard beroepenclassificatie 1992 [standardized classification of occupations 1992].

[CR56] Strøm IF, Thoresen S, Wentzel-Larsen T, Hjemdal OK, Lien L, Dyb G (2013). Exposure to life adversity in high school and later work participation: A longitudinal population-based study. Journal of Adolescence.

[CR57] Takizawa R, Danese A, Maughan B, Arseneault L (2015). Bullying victimization in childhood predicts inflammation and obesity at mid-life: A five-decade birth cohort study. Psychological Medicine.

[CR58] Takizawa R, Maughan B, Arseneault L (2014). Adult health outcomes of childhood bullying victimization: Evidence from a five-decade longitudinal British birth cohort. American Journal of Psychiatry.

[CR59] Ttofi MM, Bowes L, Farrington DP, Lösel F (2014). Protective factors interrupting the continuity from school bullying to later internalizing and externalizing problems: A systematic review of prospective longitudinal studies. Journal of School Violence.

[CR60] Ttofi MM, Farrington DP, Lösel F, Loeber R (2011). The predictive efficiency of school bullying versus later offending: A systematic/meta-analytic review of longitudinal studies. Criminal Behaviour and Mental Health.

[CR61] Turner MG, Exum ML, Brame R, Holt TJ (2013). Bullying victimization and adolescent mental health: General and typological effects across sex. Journal of Criminal Justice.

[CR62] Varhama LM, Björkqvist K (2005). Relation between school bullying during adolescence and subsequent long-term unemployment in adulthood in a Finnish sample. Psychological Reports.

[CR63] Veldman K, Bültmann U, Stewart RE, Ormel J, Verhulst FC, Reijneveld SA (2014). Mental health problems and educational attainment in adolescence: 9-year follow-up of the TRAILS study. PloS One.

[CR64] Veldman, K., Reijneveld, S. A., Ortiz, J. A., Verhulst, F. C., & Bültmann, U. (2015). Mental health trajectories from childhood to young adulthood affect the educational and employment status of young adults: Results from the TRAILS study. *Journal of Epidemiology and Community Health*. Advance Online Publication. doi:10.1136/jech-2014-204421. 10.1136/jech-2014-204421 25667302

[CR65] Verhulst FC, van der Ende J, Koot JM (1997). Handleiding voor de youth self-report (YSR).

[CR66] Viner RM, Ozer EM, Denny S, Marmot M, Resnick M, Fatusi A, Currie C (2012). Adolescence and the social determinants of health. The Lancet.

[CR67] Volk AA, Camilleri JA, Dane AV, Marini ZA (2012). Is adolescent bullying an evolutionary adaptation?. Aggressive Behavior.

[CR68] Wolke D, Copeland WE, Angold A, Costello EJ (2013). Impact of bullying in childhood on adult health, wealth, crime, and social outcomes. Psychological Science.

[CR69] Woods S, Done J, Kalsi H (2009). Peer victimisation and internalising difficulties: The moderating role of friendship quality. Journal of Adolescence.

